# Influences of dietary intake on Chinese women with gestational diabetes mellitus by inhibiting gut microbiome on plasma metabolome

**DOI:** 10.3389/fimmu.2026.1745459

**Published:** 2026-04-13

**Authors:** Qing-Xiang Zheng, Hai-Wei Wang, Li Ge, Yuzheng Lin, XiaoXia Gao, Yu Zhu, Ling Huang, Xiu-Min Jiang

**Affiliations:** 1 Fujian Maternity and Child Health Hospital College of Clinical Medicine for Obstetrics and Gynecology and Pediatrics, Fujian Medical University, Fuzhou, Fujian, China; 2 Fujian Obstetrics and Gynecology Hospital College of Clinical Medicine for Obstetrics and Gynecology and Pediatrics, Fujian Medical University, Fuzhou, Fujian, China; 3 The School of Nursing, Fujian University of Traditional Chinese Medicine, Fuzhou, China

**Keywords:** dietary intakes, gestational diabetes mellitus, gut microbiome, metabolome, microbial metabolite

## Abstract

**Background:**

Lines of evidence indicate that microbiome and its derived metabolites are implicated in gestational diabetes mellitus (GDM) etiology through the regulation of insulin resistance and inflammatory responses, and pregnant women with GDM have significant gut dysbiosis and metabolic disturbance. Although the gut microbiota and gut metabolites in pregnant women with GDM are extensively studied, the trilateral relationship between diet, gut microbiota, and plasma metabolites in patients with GDM remains unclear. Therefore, the aim of this study was to systematically analyze the associations between diet, gut microbiome, and plasma metabolome among Chinese pregnant healthy controls and patients with GDM.

**Methods:**

The study is a prospective cohort study conducted at two maternal and child hospitals in China from 8 October 2021 to 31 December 2022. We compared the daily dietary intake, microbial compositions, and plasma metabolic signatures of 173 patients with GDM and 47 pregnant healthy individuals. A food frequency questionnaire was used to investigate the dietary intake of pregnant healthy controls and patients with GDM. 16S rRNA sequencing and liquid chromatography–tandem mass spectrometry (LC-MS/MS) were used to sequence the gut microbiome and plasma metabolome, respectively.

**Results:**

We found that women with GDM had higher intakes of whole grains, red meat, poultry, and eggs compared with normal pregnant women. Women with GDM had lower amounts of *Klebsiella*, *Lactiplantibacillus*, and *Sphingomonas*, and higher amounts of *Desulfovibrio*; they also had higher amounts of D-mannose, D-ribose, homo-L-arginine, and norophthalmic acid in plasma. Moreover, whole grains negatively influenced *Sphingomonas*, *Klebsiella*, and *Lactiplantibacillus*; red meat had a negative influence on *Sphingomonas*; and eggs had a positive impact on *Desulfovibrio*; these gut microbiota affected D-mannose, D-ribose, homo-L-arginine, and norophthalmic acid.

**Conclusion:**

Overall, this study provided information about the influences of dietary intake on Chinese women with GDM by inhibiting gut microbiome on plasma metabolome, and their interactions play vital roles in GDM pathogenesis. These findings may be useful for patients with GDM in terms of dietary counseling and glucose control during pregnancy.

## 1 Introduction

Gestational diabetes mellitus (GDM) is characterized by hyperglycemia and insulin resistance in the second or third trimester of pregnancy (1) and has become a leading pregnancy complication worldwide (2). The prevalence of GDM has been skyrocketing in decades (3). Approximately 3.52 million pregnant women were affected by GDM globally (4). GDM could cause several adverse maternal–infant outcomes (5). Moreover, women with GDM (6) and their offspring (7) have higher risks of abnormal glucose tolerance and reduced β-cell function (8) in later life. Therefore, measures of preventing and treatment of GDM are greatly needed (2).

Studies found that pre-pregnancy and pregnancy dietary patterns were closely related to the occurrence and development of GDM (9, 10). Healthy dietary choices influence gut microbiota composition and induce the production of beneficial metabolites in plasma (11). With recent lifestyle innovations, high-fat/high-sugar diets had altered the genetic composition and metabolic activity of the host gut microbiome (12). This highlights the essential associations among healthy diet, gut microbiota, and plasma metabolism in the development and treatment of GDM.

Gut microbiota balance and structural diversity are important to maintain the normal energy metabolism and to resist the invasion of pathogenic microorganisms in the human host (13). Lines of evidence indicate that microbiome and its derived metabolites are implicated in GDM etiology (14) through the regulation of insulin resistance and inflammatory responses (15, 16); women with GDM have significant gut dysbiosis (17–19) and metabolic disturbance (1, 20, 21). Therefore, microbiome and its derived metabolites are potential effective measures to prevent and treat GDM. Previously, we found that *Lactobacillus* and *Bifidobacterium* probiotics supplements could reduce the fasting blood glucose level of GDM rats by restoring the diversity of gut microbiota (22) and alleviate the pathology of GDM through multiple metabolism pathways in rats (23). However, the effects of probiotics microbiome on plasma metabolomics of patients with GDM are not clear.

Gut microbiota and metabolites are influenced by many factors such as diet, lifestyle, and individual genetic background (24). The microbiome partly but significantly affects the individual metabolism and responds to changes in dietary habits (25, 26). Although the gut microbiota and gut metabolites in patients with GDM are extensively studied (1, 21), the trilateral relationship between diet, gut microbiota, and plasma metabolites in patients with GDM remains unclear. In this study, we compared the daily dietary intake, microbial compositions, and plasma metabolic signatures of Chinese pregnant healthy controls and patients with GDM. This integration of multi-level data will systematically reveal these interrelated associations. Our findings will provide useful information for women with GDM in dietary counseling and novel strategies to prevent and treat GDM during pregnancy.

## 2 Method

### 2.1 Participants

The study is a prospective cohort study conducted at the Fujian Maternity and Child Health Hospital and the Fujian Obstetrics and Gynecology Hospital in China from 8 October 2021 to 31 December 2022. The inclusion criteria of participants are as follows: (1) A single pregnancy conceived naturally; (2) the diagnostic criteria for 75-g oral glucose tolerance test (OGTT) were obtained from the “Diagnosis Guidelines for Gestational Diabetes Mellitus (2014)”; (3) pregnant women aged 20 to 35 years; (4) having regular routine prenatal examinations; (5) no severe pregnancy complications or comorbidities, such as preeclampsia and eclampsia; and (6) pregnant women voluntarily consented to participate in this study, as well as to both fecal and blood sample collection. Meanwhile, participant exclusion criteria included the following: (1) Pregnant women with a history of diabetes mellitus before pregnancy, hypertension, or depression, or those with congenital heart disease and hepatic or chronic enteritis; (2) use of corticosteroids before pregnancy and during pregnancy; (3) concurrent acute gastroenteritis within the past month; (4) maternal with incomplete prenatal examination and delivery records; and (5) no impairments in consciousness and cognition. The ethics review board of hospitals has approved the study (No. 2021KLRD0645 and No. 2021KR041). Meanwhile, this study was registered with the Chinese Clinical Trial Registry (registration no. ChiCTR2500104951) at https://www.chictr.org.cn/showproj.html?proj=276358. All pregnant women signed informed consent forms before questionnaire collection. The informed consent clarified that all participants were required to voluntarily fill in questionnaires and provide fecal and blood samples for 24 to 28 gestational weeks. Furthermore, all of them were offered a standardized OGTT during the 24- to 28-gestational-week period. Women with GDM were diagnosed by an obstetrician with the following criteria (27): fasting plasma glucose (OGTT0h) ≥ 5.1 mmol/L, 1 h plasma glucose (OGTT1h) ≥ 10.0 mmol/L, or 2 h plasma glucose (OGTT2h) ≥ 8.5 mmol/L. The inclusion flow of the study population is shown in **Supplementary Figure 1**. Moreover, our data collection period was in the post-pandemic era. Our research institute was located in a low-risk area with minimal exposure to COVID-19, allowing for unrestricted mobility. Additionally, all participants maintained normal physiological conditions throughout the data collection period and had not been infected with COVID-19.

### 2.2 Demographic and dietary intake data collection

A questionnaire was used to collect demographic data and dietary intake information. Dietary intake was investigated using a food frequency questionnaire (FFQ), which has been proven as a valid and reliable assessment tool for estimating the diet intake of Chinese pregnant women (28). FFQ as a “data-driven method” utilized dietary data from a study population to summarize dietary patterns and analyze the relationship between dietary patterns and disease risk. The FFQ comprises 61 food items, which cover more than 200 food species. Except for drinks and oils, other food items were divided into 16 dietary intake groups with similar species characteristics or nutrient profiles. Six investigators investigated the FFQ via face-to-face interview. All investigators received systematic training via a visual food intake booklet, and then they were instructed by nutrition experts on how to successfully use the FFQ. Each participant was required to recall the food intake frequency and average consumption of each food item for the last 3 months. According to Li et al. (29), the original categories of the intake frequency were adjusted into six grades: “never” = 0, “1–3 times per 4 weeks” = 1, “1–3 times per week” = 2, “4–6 times per week” = 3, “1–2 times per day” = 4, and “more than two times per day” = 5. Then, we calculated the daily intake of each food item (in grams), which was the newly assigned frequency multiplied by consumption per serving and dividing the product by 28 for standard portions (29).

### 2.3 ELISA of plasma parameters

Plasma parameters including blood glucose, insulin, and lipopolysaccharide (LPS), and lipid factors including total cholesterol (T-CHO), triglyceride (TG), low-density lipoprotein cholesterol (LDLC), and high-density lipoprotein cholesterol (HDLC) were measured using enzyme-linked immunosorbent assay (ELISA) kits. The homeostasis model assessment-estimated insulin resistance (HOMA-IR) was also further calculated.

### 2.4 Fecal collection and 16S rRNA sequencing

Fecal samples (approximately 200 mg) from participants were collected. 16S rRNA sequencing and data processing were described in our previous paper (22). Briefly, fecal DNA was extracted by the PowerSoil DNA Isolation Kit (Qiagen) and used as template to amplify the V3 and V4 regions of the 16S rRNA gene. The AMpure XP Kit (Beckman Coulter, CA, USA) was used to purify the PCR products, and the purified products were barcoded, pooled to construct the sequencing library, and then sequenced using the Illumina HiSeq platform (Illumina) through the paired-end sequencing strategy. A total of 17,517,597 clean reads were clustered into OTUs after removing low-quality sequences via Trimmomatic and UCHIME software. A total of 10,533 OTUs in the GDM group and 3,481 OTUs in the normal group were chosen from the RDP database (confidence threshold 0.8) with 97% similarity. In this study, we aimed to concern broad the microbial compositions so we selected 97% OTU clustering. Moreover, alpha diversity was analyzed by Mothur software to explore the gut microbiota richness and diversity, and beta diversity was analyzed by QIIME software to evaluate the gut microbiota differences in species complexity among samples. Alpha diversity assessed by the Chao1 index was compared using the Mann–Whitney *U* test. Beta diversity differences in microbial community composition were illustrated by PCoA and analyzed by PERMANOVA. The relative abundance of phyla and genera was compared by the Wilcoxon rank-sum test or the analysis of variance (ANOVA) test. Raw sequence data are available in the Sequence Read Archive under BioProject accession number PRJNA1052755.

### 2.5 Blood plasma collection and LC-MS/MS analyses

All blood samples from participants were collected on the day of prenatal examination. Blood samples were immediately centrifuged to separate plasma. Liquid chromatography–tandem mass spectrometry (LC-MS/MS) analyses were performed via an ultrahigh-performance liquid chromatography system (Vanquish, Thermo Fisher Scientific) with a UPLC BEH amide column (2.1 × 100 mm, 1.7 µm) coupled to a Q Exactive HF-X mass spectrometer (Orbitrap MS, Thermo Fisher Scientific) in both positive and negative modes simultaneously. LC-MS/MS analysis was strictly based on the manufacturer’s instructions. The metabolite annotation was performed via an in-house MS2 database (BiotreeDB), and the cutoff for metabolite annotation was set at 0.3.

OPLS-DA was used to distinguish metabolomic differences. Plasma metabolites between two groups had a significant difference when the projection of the first principal component of OPLS-DA > 1, the absolute fold change is > 1 or < 0.5, and *p* < 0.05. The pathway analysis for metabolites was calculated by MetaboAnalyst to predict the enriched pathway of significant different metabolites. The hierarchical clustering analysis of metabolites between the two groups was conducted using the R software pheatmap function. The correlations between plasma parameters, diet, gut microbiota, and metabolites were calculated by the Spearman’s test via the psych package in the R software.

### 2.6 Data collection and data detection

All participants voluntarily consented to data collection and data detection. The follow-up period was completed at 28^+6^ gestational weeks after the diagnosis of GDM, and dietary data, fecal samples, and blood samples were collected during this period. All fecal samples and blood samples were stored in a −80°C refrigerator from 8 October 2021 to 31 December 2022. After that, all fecal samples and blood samples were processed or sequenced in the same batch.

### 2.7 Statistical analysis

Means ± SD, medians (IQR), or proportions were calculated for participant characteristics. The demographic data and plasma parameters between the GDM group and the normal group were analyzed by the χ^2^ test, Fisher exact test, or *t*-test. For dietary intake data, the difference in dietary intake between the two groups was analyzed by non-parametric test. Spearman correlation analyses were used to analyze associations among plasma parameters, diet, gut microbiota, and plasma metabolites. Statistical analysis and related figures plotted in this study were conducted using R software (version 4.3.1), SPSS (version 27.0), and GraphPad Prism (version 9.5). *p* < 0.05 indicated a significant difference.

## 3 Results

### 3.1 Characteristics of participants

A total of 648 pregnant women were originally involved in this study (436 women with GDM and 212 normal pregnant women). Among them, 321 pregnant women voluntarily consented to both fecal and blood sample collection (191 women with GDM and 130 normal pregnant women). Finally, after both fecal and blood sample quality testing and data integrity testing, 173 women with GDM and 47 normal pregnant women were finally analyzed (**Supplementary Figure 1**). Since pregnant women in the normal group might be in a healthy and well-conditioned state, the lack of dietary and clinical data was more significant. The final sample size was included to the greatest extent possible. Participants between the two groups were statistically different in gestational weeks (**Table 1**). The values of OGTT, glycosylated hemoglobin, and family history of diabetes mellitus in the GDM group were significantly higher in the normal group (*p* < 0.05) (**Table 1**). Furthermore, compared to normal pregnant women, the concentrations of blood glucose, insulin, and LDL-C increased in women with GDM (*p* < 0.05) (**Table 1**). However, the concentrations of HOMA-IR and T-CHO in the GDM group were lower than those in the normal group (*p* < 0.05) (**Table 1**). All characteristics of participants are presented in **Table 1**.

**Table 1:** Characteristics of study participants and comparison of plasma parameters and diet intakes between the GDM group and the control group (*n* = 220).

Variables		GDM group (*n* = 173)	Control group (*n* = 47)	*p*
Characteristics of study participants				
Age (years)		31.35 ± 4.15	31.17 ± 3.70	0.785
Family monthly income per capita (RMB)	<3,000	4 (1.82%)	2 (0.90%)	0.353
	3,000–5,999	51 (23.18%)	16 (7.27%)	
	6,000–8,999	66 (30.00%)	21 (9.55%)	
	9,000–11,999	31 (14.09%)	3 (1.36%)	
	≥12,000	21 (9.55%)	5 (2.28%)	
Gestational weeks		26.71 ± 0.18	27.98 ± 0.30	0.001*
Pre-pregnancy body mass index (kg/m^2^)		22.65 ± 3.20	22.98 ± 2.79	0.524
Gravidity (time)	1	73 (33.18%)	19 (8.64%)	0.961
	2	59 (26.82%)	16 (7.27%)	
	3 or more than 3	41 (18.64%)	5 (5.45%)	
Parity (time)	0	107 (48.64%)	25 (11.36%)	0.551
	1	58 (26.36%)	19 (8.64%)	
	2	0 (0.00%)	3 (1.36%)	
Cesarean section number (time)	0	144 (65.46%)	40 (18.18%)	0.791
	1	27 (12.27%)	6 (2.74%)	
	2	2 (0.90%)	1 (0.45%)	
75 g oral glucose tolerance test (OGTT) (mean ± SD)	OGTT0h	4.87 ± 0.52	4.47 ± 0.23	<0.001*
	OGTT1h	10.01 ± 1.81	7.24 ± 1.40	<0.001*
	OGTT2h	8.48 ± 1.75	6.51 ± 1.03	<0.001*
Glycosylated hemoglobin (%)		4.70 ± 1.58	4.23 ± 1.10	<0.001*
Family history of diabetes mellitus (*N*)	Yes	63 (28.64%)	7 (3.18%)	0.005*
	No	110 (50.00%)	40 (18.18%)	
Plasma parameters level (mean ± SD)				
Blood glucose (mg/mL)		0.59 ± 0.17	0.51 ± 0.20	0.017*
Insulin (pmol/L)		404.52 ± 407.13	260.40 ± 280.46	0.027*
HOMA-IR		7.35 ± 10.15	12.99 ± 15.66	0.033*
LPS (ng/L)		559.88 ± 393.45	512.57 ± 404.16	0.533
T-CHO (mmol/L)		6.63 ± 1.57	8.44 ± 2.89	<0.001*
TG (mmol/L)		2.41 ± 0.50	2.46 ± 1.01	0.777
LDL-C (mmol/L)		11.80 ± 14.85	6.64 ± 8.93	0.023
HDL-C (mmol/L)		2.48 ± 0.89	2.37 ± 0.49	0.404
Diet intakes (median [IQR])				
Rice and wheat products (g/day)		15 (9.55, 22.68)	13.57 (8.82, 20)	0.211
Whole grains (g/day)		5.71 (1.82, 16.62)	4.36 (0, 9.14)	0.041*
Beans and bean products (g/day)		21.61 (8.75, 32.14)	17.86 (9.64, 26.79)	0.306
Leafy and cruciferous vegetables (g/day)		21.43 (11.07, 28.57)	17.86 (11.43, 29.14)	0.511
Root vegetables (g/day)		6.96 (3.48, 11.96)	5.36 (2.5, 9.46)	0.15
Mushrooms and algae (g/day)		17.86 (9.29, 27.07)	15.71 (10.71, 21.79)	0.594
Melon and solanaceous vegetables (g/day)		3.57 (1.43, 5.71)	2.14 (0.71, 5.71)	0.072
Fruits (g/day)		50.54 (37.61, 71.89)	49.64 (35.86, 67.5)	0.718
Dairy products (g/day)		35.71 (28.57, 45.89)	35.71 (28.57, 42.86)	0.735
Red meat (g/day)		9.25 (5.54, 15)	5.71 (4.29, 12.14)	0.01*
Poultry (g/day)		3.57 (0.80, 7.14)	1.79 (0.00, 3.57)	0.02*
Animal organ and blood products (g/day)		0.00 (0.00, 1.25)	0.00 (0.00, 1.79)	0.642
Freshwater fishes (g/day)		10.71 (7.14, 17.79)	8.57 (5, 21.43)	0.676
Seafood (g/day)		10.71 (7.14, 17.79)	8.57 (5.00, 21.43)	0.676
Eggs (g/day)		8.57 (7.14, 10.00)	8.57 (7.71, 8.57)	0.006*
Nuts (g/day)		1.07 (0.00, 2.14)	1.07 (0.00, 3.57)	0.496

*p < 0.05.

### 3.2 Diet profiling of women with GDM and normal pregnant women

**Figure 1:**
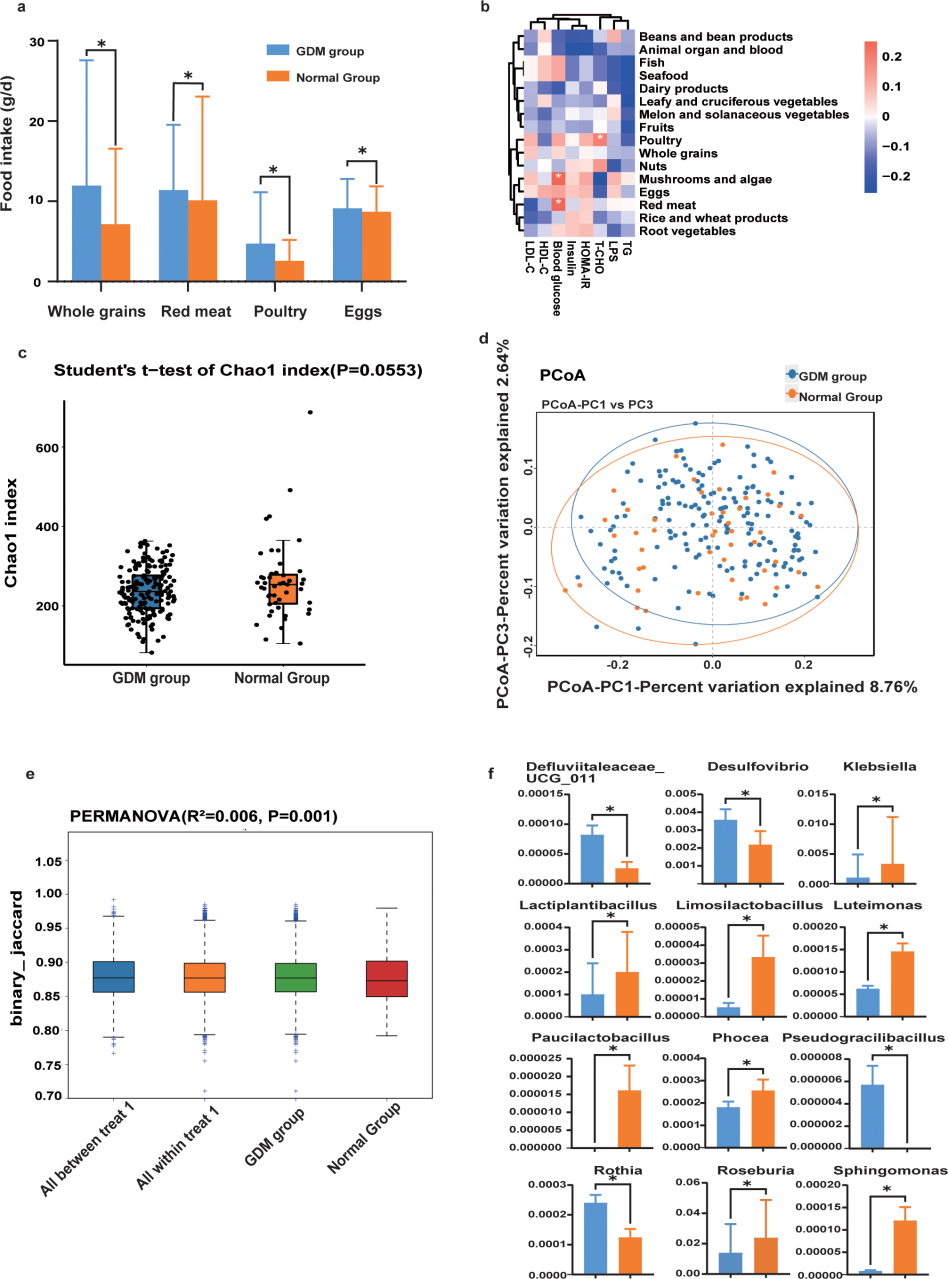
Diet intake and gut microbial characteristics. **(a)** Difference in whole grain, red meat, poultry, and egg intake between the GDM group and the normal group (*p < 0.05). **(b)** Spearman’s correlation analysis of plasma parameters and diet. The *r*-values were denoted with graduated colors, and red and blue grids indicated positive and negative correlations, respectively (*p < 0.05). **(c)** Alpha diversity of the gut microbiome between the GDM group and the normal group. **(d)** PCoA, based on unweighted UniFrac distance at the OTU level showing a discriminative trend of microbial composition between women with GDM and normal pregnant women. **(e)** PERMANOVA analysis between the GDM group and the normal group (*R*^2^ = 0.006, *p* = 0.001). **(f)** Key altered gut microbiota abundances in GDM (*p < 0.05).

The FFQ investigation result showed that the dietary intake between women with GDM and normal pregnant women was similar, and they tended to eat more fruits, dairy products, vegetable, and meat to ensure the availability of fetal nutrition (**Table 1**). Among 16 dietary intakes, whole grain, red meat, poultry, and egg intakes of women with GDM were significantly higher than those of normal pregnant women (**Figure 1a**). Moreover, blood glucose was positively associated with red meat (*p* < 0.05), and T-CHO was also positively related to poultry (*p* < 0.05) (**Figure 1b**).

### 3.3 Microbiota was different between women with GDM and normal pregnant women

A total of 220 fecal samples were analyzed via 16S rRNA sequencing. There was no significant difference in alpha diversity (Chao1 index: *p* = 0.0553) between the GDM group and the normal group (**Figure 1c**). PCoA results showed that there was no significant separation trend in these two dimensions between the two groups (**Figure 1d**); however, PERMANOVA indicated that women with GDM had a significant reduction of beta diversity compared with normal pregnant women (*R*^2^ = 0.006, *p* = 0.001) (**Figure 1e**). These results suggested that the microbial community structure in pregnant women with GDM was highly concentrated, with minimal inter-individual variation; in contrast, the microbial community diversity in normal pregnant women was significantly higher, exhibiting marked individual differences. Furthermore, there were 109 significantly changed gut microbial taxa at the genus level between the two groups (*p* < 0.05) (**Supplementary Table 1**). Women with GDM had lower amounts of *Roseburia*, *Klebsiella*, *Lactiplantibacillus*, and *Sphingomonas*, among others, and a higher amount of *Desulfovibrio* and *Rothia* (**Figure 1f**).

### 3.4 Plasma metabolites were different between the GDM group and the normal group

**Figure 2:**
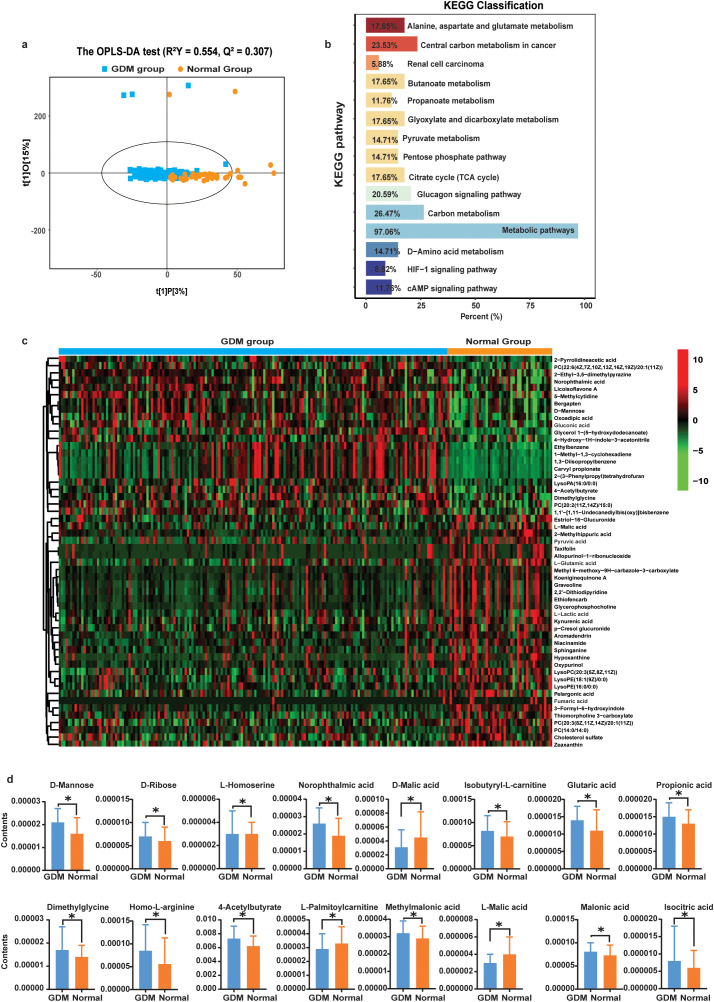
Plasma metabolite features between the GDM group and the normal group. **(a)** OPLS-DA of the plasma metabolomic profiles between the two groups. **(b)** Plasma metabolites signaling pathways in the KEGG database (the horizontal axis represents the percentage of annotated differential metabolites in a specific pathway relative to the total number of annotated differential metabolites, while the vertical axis indicates the name of the enriched KEGG metabolic pathway). **(c)** Hierarchical clustering analysis of 54 metabolites between women with GDM and normal pregnant women (red means positive relation, while green means negative relation). **(d)** Key altered plasma metabolite abundances in relation to GDM (*p < 0.05).

A total of 220 blood samples were used for the plasma metabolome study. A total of 657 metabolites were detected across all samples. The OPLS-DA analysis showed that plasma metabolites were significantly different between the GDM group and the normal group (*R*^2^*Y* = 0.554, Q2 = 0.307) (**Figure 2a**). Based on the criteria of *p* < 0.05, 117 metabolites were different between the two groups (**Supplementary Table 2**), and the KEGG enrichment analysis results of differential metabolites were mainly enriched in metabolic pathways (97.06%); carbon metabolism (26.47%); central carbon metabolism in cancer (23.53%); glucagon signaling pathway (20.59%); alanine, aspartate, and glutamate metabolism (17.65%); butanoate metabolism (17.65%); glyoxylate and dicarboxylate metabolism (17.65%); and citrate cycle (TCA cycle) (17.65%) (**Figure 2b**). A total of 54 metabolites were different based on the criteria of *p* < 0.01 (**Figure 2c**). Among these significant metabolites, women with GDM had higher amounts of D-mannose, D-ribose, homo-L-arginine, and norophthalmic acid, and had lower amounts of D-malic acid, L-palmitoylcarnitine, and L-malic acid (**Figure 2d**).

### 3.5 Associations among plasma parameters, diet, gut microbiota, and plasma metabolites

Next, we analyzed associations among plasma parameters, diet, gut microbiota, and plasma metabolites. Firstly, the associations between plasma parameters and diet are shown in **Figure 1b**. Secondly, the associations between plasma parameters and gut microbiota showed that blood glucose was positively associated with *Desulfovibrio*, while it was negatively associated with *Bacteroides* (*p* < 0.05) (**Supplementary Figure 2a**). Thirdly, the associations between plasma parameters and plasma metabolites indicated that blood glucose, insulin, and HOMA-IR were all positively associated with D-mannose, L-homoserine, D-galactose, D-ribose, and glutaric acid, while blood glucose and HOMA-IR were negatively correlated with L-lactic acid and L-malic acid (*p* < 0.05) (**Supplementary Figure 2b**). Fourthly, we found that 29 gut microbiota taxa were significantly correlated with dietary intakes (**Supplementary Figure 2c**). Specifically, whole grains were significantly associated with *Rothia*, *Klebsiella*, *Sphingomonas*, and *Lactiplantibacillus*; red meat was significantly correlated with *Phocea* and *Pseudogracilibacillus*; poultry was significantly associated with *Paucilactobacillus*; and eggs were significantly correlated to *Desulfovibrio*, *Defluviitaleaceae_UCG_011*, and *Gemmatimonas* (**Supplementary Figure 2d**). Fifthly, we also found that 25 metabolites including amino acids, glucuronide, short-chain fatty acid, fatty acids, and organic acid were significantly associated with dietary intakes (**Supplementary Figure 2e**). Among these, whole grains were positively associated with homo-L-arginine; red meat had a positive correlation with gluconolactone and ethylbenzene, and had a negative association with fumaric acid; poultry was positively associated with homo-L-arginine and isobutyryl-L-carnitine, and negatively associated with 3-formyl-6-hydroxyindole and Fumaric acid; eggs were positively correlated with p-Cresol sulfate (**Supplementary Figure 2f**). Finally, the changed metabolites of GDM may be perturbed by gut microbiota during GDM pathogenesis (23). Spearman’s correlation results indicated that 26 gut microbial taxa at the genus level were significantly correlated with 34 metabolites (**Supplementary Figure 3**). In particular, *Sphingomonas*, *Klebsiella*, and *Lactiplantibacillus* displayed strong correlations with the metabolism of amino acids, glucuronide, fatty acids, and organic acids (**Supplementary Figure 3**).

### 3.6 Interrelations among diet, GDM-related gut microbiota, and plasma metabolites

**Figure 3:**
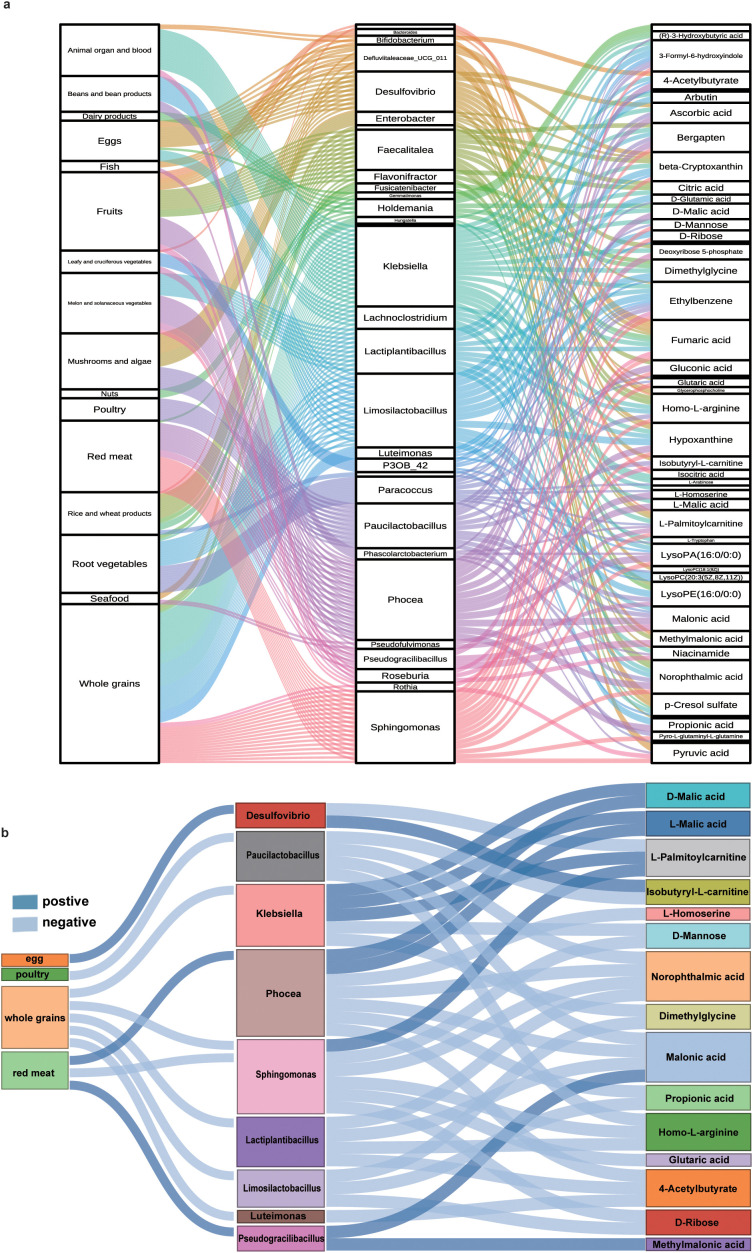
Interrelations among diet, GDM-related gut microbiota, and metabolites. **(a)** Interrelations among diet, GDM-related gut microbiota, and metabolites. **(b)** Interrelations among whole grain, red meat, poultry, and egg intake; GDM-related gut microbiota; and metabolites (dark blue means positive relation, while light blue means negative relation).

Finally, we conducted analyses to comprehensively determine the interrelations among diet and gut microbiota at the genus level and plasma metabolites via Sankey diagrams. Firstly, we showed that dietary intakes had comprehensive influences on gut microbiota, and then affected metabolites (**Figure 3a**). Among 16 dietary intakes, whole grains, red meat, and fruit mainly affected gut microbiota such as *Klebsiella*, *Sphingomonas*, *Lactiplantibacillus*, and *Desulfovibrio* (**Figure 3a**). Then, gut microbiota further influenced metabolites like norophthalmic acid, homo-L-arginine, and L-palmitoylcarnitine (**Figure 3a**). And then, we further explore the positive or negative correlations among significant difference diet, related changed gut microbiota and plasma metabolites via another Sankey diagram. We found that whole grains positively influenced *Sphingomonas*, *Klebsiella*, *Limosilactobacillus*, *Lactiplantibacillus*, and *Luteimonas*; red meat had a positive effect on *Phocea* and *Pseudogracilibacillus* and had a negative influence on *Sphingomonas*; eggs positively affected *Desulfovibrio*; poultry negatively affected *Paucilactobacillus*; these nine gut microbial taxa affected 15 metabolites including glucuronides (D-mannose and D-ribose), amino acids (homo-L-arginine, norophthalmic acid, dimethylglycine, and L-homoserine), fatty acids (4-acetylbutyrate, isobutyryl-L-carnitine, L-palmitoylcarnitine, and D-malic acid), and organic acids (glutaric acid, malonic acid, L-malic acid, methylmalonic acid, and propionic acid) (**Figure 3b**).

## 4 Discussion

### 4.1 Principal findings

In this study, we systematically analyzed the associations among diet, gut microbiome, and plasma metabolome among Chinese pregnant healthy controls and patients with GDM. We found that women with GDM had higher intakes of whole grains, red meat, poultry, and eggs compared with normal pregnant women. It seemed that women with GDM tended to have an animal-based diet. However, studies suggested that a plant-based diet presents healthier choices to prevent disease than an animal-based diet (30, 31). Based on these observations, a Mediterranean diet was a nutritionally recommended diet for women GDM, which included high-level consumption of cereals, fruit, vegetables, and legumes (11).

Moreover, we also found that women with GDM had lower amounts of *Klebsiella*, *Lactiplantibacillus*, and *Sphingomonas*, and higher amounts of *Desulfovibrio* and *Rothia*. Similarly, Crusell et al. showed that *Desulfovibrio* and *Rothia* at the genus level in the GDM cohort had a higher abundance than those in the normal cohort (17). *Desulfovibrio* was also enriched in patients with type 2 diabetes (32, 33). Moreover, *Lactobacillus* was associated with higher weight gain during pregnancy (17). Lactic acid bacteria (*Lactobacillus acidophilus*, *Lactobacillus delbrueckii*, *Lactiplantibacillus plantarum*, and *Limosilactobacillus fermentum*) were often used as probiotics (34). The gut microbiota modulation by lactic acid bacteria was widely used to alleviate overweight and obesity and mitigate metabolic diseases such as diabetes (34, 35). Kuang et al. also suggested that *Klebsiella variicola* were enriched in women without GDM (19). Wang et al. found that in comparison with healthy pregnant controls, individuals with GDM showed a higher abundance of bacterial OTUs belonging to the family Lachnospiraceae, and a lower abundance of OTUs from the families Enterobacteriaceae and Ruminococcaceae, and four metabolites in feces and fifteen in urine that differentiate GDM from pregnant healthy controls, which was mainly involved in carbohydrate and amino acid metabolism (21). Studies suggest pregnant women or rats with GDM have gut dysbiosis. Previous studies have also demonstrated that gut microbiota plays a pivotal role in regulating insulin resistance and inflammatory responses in GDM (15, 16). However, the detailed functions of those gut microbiome should be further studied.

Furthermore, women with GDM had higher amounts of D-mannose, D-ribose, homo-L-arginine, and norophthalmic acid, and had lower amounts of D-malic acid, L-palmitoylcarnitine, and L-malic acid in plasma. Plasma metabolites were mainly enriched in glucose, amino acid, and lipid metabolism. These results were similar to previous studies (1, 21, 23). Among them, D-mannose was a natural monosaccharide related to glucose. A study had shown that plasma D-mannose levels were significantly associated with insulin resistance (36). This might be related to the fact that GDM was closely related to insulin resistance and pancreatic β-cell dysfunction. Meanwhile, GDM was a glucose metabolism disorder occurring during pregnancy, accompanied by varying degrees of lipid and amino acid metabolism abnormalities. Ye et al. found that women with GDM exhibited a marked increase in L-alpha-aminobutyric acid and 2-hydroxybutyric acid of plasma metabolites, but a decrease in allantoin, methionine sulfoxide, dopaminergic synapse, and dopamine (1). However, those plasma metabolites were not detected in our study. Such consistency highlighted the dynamics of plasma metabolites in different cohorts, and a plasma metabolite analysis should be performed in more diverse and larger populations with GDM.

The gut microbiome and plasma metabolome could rapidly respond to the altered diet (26). In this study, we revealed the diet-associated gut microbiome and plasma metabolome among women with GDM. We showed that whole grains were significantly associated with *Rothia*, *Klebsiella*, *Sphingomonas*, and *Lactiplantibacillus*; red meat was significantly associated with *Phocea* and *Pseudogracilibacillus*, poultry was significantly associated with *Paucilactobacillus*, and eggs were significantly correlated with *Desulfovibrio*, *Defluviitaleaceae_UCG_011*, and *Gemmatimonas*. Furthermore, whole grains were significantly associated with ethylbenzene; red meat was significantly correlated with fumaric acid, ethylbenzene, and gluconolactone; poultry was significantly associated with fumaric acid, isobutyryl-L-carnitine, and homo-L-arginine; and eggs were significantly correlated with p-Cresol sulfate. Those results were consistent with the finding that meaningful alterations of the gut microbiome were associated with alterations in diet, primarily affected by dietary fiber consumption from vegetables, fruits, and other plants (37), and dietary fiber fermentation was identified to be associated with GDM status and host glucose metabolism (20).

In revealing the trilateral associations among diet, gut microbiome, and plasma metabolome among women with GDM, we further found that whole grains and red meat had an interrelated and negative influence on *Sphingomonas*, and then *Sphingomonas* had a positive effect on L-palmitoylcarnitine and had negative impacts on D-mannose, D-ribose, homo-L-arginine, norophthalmic acid, and glutaric acid. Moreover, whole grains negatively influenced *Klebsiella* and *Lactiplantibacillus*, and eggs positively impacted *Desulfovibrio*; these gut microbiota affected D-mannose, D-ribose, homo-L-arginine, and norophthalmic acid. The interactions of diet, gut microbiome, and plasma metabolome in GDM pathogenesis were previously unreported and should be further studied.

### 4.2 Strengths and limitations

To our best knowledge, this was the first analysis of the trilateral associations among dietary intakes, gut microbiome, and plasma metabolome among Chinese women with GDM. Moreover, we addressed many microbial and metabolic biomarkers of GDM, such as *Klebsiella*, *Lactiplantibacillus*, *Sphingomonas*, and *Desulfovibrio* at the genus level, and D-mannose, D-ribose, homo-L-arginine, and norophthalmic acid, among others. However, all pregnant women in this study were Chinese; further studies with a more diverse and larger population need to be performed to confirm and explain these findings. This study is based on observational data of dietary intakes; interpretation of the association between diet, gut microbiome, and metabolome should be made with caution, and future intervention and experimental studies that focus on specific diets and microbial genomic capacities and their metabolome are essential to confirm causality.

## 5 Conclusion

Overall, this study provided information about the influences of dietary intake on Chinese women with GDM by inhibiting gut microbiome on plasma metabolome, and suggested that interactions between diet, gut microbiome, and plasma metabolome play vital roles in GDM pathogenesis. These findings may be useful for patients with GDM in terms of dietary counseling and glucose control during pregnancy. Moreover, our results suggest that diet, GDM-related gut microbiota, and plasma metabolites are promising targets for GDM prevention and treatment.

## Data Availability

The datasets generated during sequencing of fecal DNA samples have been deposited in NCBI Sequencing Read Archive (accession number: PRJNA1052755). Other datasets from the current study are available from the corresponding author upon request.
