# Supplementary material for: Influences of dietary intake on Chinese women with gestational diabetes mellitus by inhibiting gut microbiome on plasma metabolome
Source: Front Immunol. 2026 Apr 13;17:1745459. doi: 10.3389/fimmu.2026.1745459 (PMC13110953; doi:10.3389/fimmu.2026.1745459)
Supplement: Supplementary file 1 [file Supplementaryfile1.docx]

Supplementary Material

# Supplementary Figures and Tables

For more information on Supplementary Material and for details on the different file types accepted, please see [here](https://www.frontiersin.org/guidelines/author-guidelines#supplementary-material).

## Supplementary Tables

**Supplementary Table S1** The 109 significantly changed gut microbiota at genus level in GDM group vs. Normal group (n=220).

| Genus | GDM(mean) | GDM(se) | NOR(mean) | NOR(se) | p | p(FDR) |
| --- | --- | --- | --- | --- | --- | --- |
| Achromobacter | 0 | 0 | 4.35E-06 | 2.98E-05 | 0.028 | 0.288 |
| Acidibacter | 1.48E-07 | 1.48E-07 | 2.16E-06 | 1.51E-06 | 0.016 | 0.285 |
| Acidovorax | 0 | 0 | 6.54E-06 | 4.49E-05 | 0.028 | 0.283 |
| Aeromicrobium | 0 | 0 | 8.19E-07 | 5.61E-06 | 0.028 | 0.279 |
| Aestuariibacter | 0 | 0 | 5.42E-07 | 3.72E-06 | 0.028 | 0.275 |
| Agathobacter | 0.012346073 | 0.026505012 | 0.015449049 | 0.0251926 | 0.050 | 0.224 |
| Alistipes | 0.011539135 | 0.012319795 | 0.005850816 | 0.005522231 | 0.001 | 0.024 |
| Aminobacterium | 0 | 0 | 8.09E-07 | 5.55E-06 | 0.028 | 0.271 |
| Anaerotruncus | 0.000905386 | 0.005529271 | 0.000345289 | 0.000672443 | 0.011 | 0.166 |
| Aquabacterium | 0 | 0 | 1.65E-06 | 1.13E-05 | 0.028 | 0.267 |
| Arcanobacterium | 0 | 0 | 1.66E-06 | 1.14E-05 | 0.028 | 0.263 |
| Blastomonas | 0 | 0 | 1.09E-06 | 7.46E-06 | 0.028 | 0.260 |
| Blautia | 0.025341376 | 0.001776587 | 0.018375204 | 0.001682102 | 0.049 | 0.285 |
| Bradyrhizobium | 0 | 0 | 3.52E-06 | 2.64E-06 | 0.011 | 0.229 |
| Bryobacter | 2.95E-07 | 2.08E-07 | 3.56E-06 | 2.55E-06 | 0.019 | 0.285 |
| Burkholderia_Caballeronia_Paraburkholderia | 0 | 0 | 1.09E-06 | 7.47E-06 | 0.028 | 0.256 |
| Butyricicoccus | 0.00338786 | 0.000208338 | 0.00440513 | 0.000506479 | 0.035 | 0.285 |
| Caenimonas | 0 | 0 | 3.82E-06 | 2.62E-05 | 0.028 | 0.253 |
| Candidatus_Actinomarina | 0 | 0 | 5.41E-07 | 3.71E-06 | 0.028 | 0.249 |
| Candidatus_Alysiosphaera | 0 | 0 | 1.11E-06 | 7.74E-07 | 0.006 | 0.180 |
| Cellulosilyticum | 2.49E-05 | 1.51E-05 | 0.000148439 | 9.32E-05 | 0.029 | 0.285 |
| Chloronema | 0 | 0 | 5.46E-07 | 3.74E-06 | 0.028 | 0.246 |
| Citrifermentans | 0 | 0 | 5.45E-07 | 3.74E-06 | 0.028 | 0.243 |
| Clostridioides | 0 | 0 | 1.35E-05 | 9.24E-05 | 0.028 | 0.240 |
| Curvibacter | 0 | 0 | 3.02E-06 | 2.07E-05 | 0.028 | 0.237 |
| Defluviitaleaceae_UCG_011 | 8.19E-05 | 0.000208784 | 2.56E-05 | 7.46E-05 | 0.020 | 0.271 |
| Desulfovibrio | 0.003569349 | 0.007885337 | 0.002175552 | 0.0052564 | 0.046 | 0.213 |
| DMER64 | 0 | 0 | 8.23E-07 | 5.64E-06 | 0.028 | 0.234 |
| Dorea | 0.002450777 | 0.000231512 | 0.005195804 | 0.002510025 | 0.047 | 0.285 |
| DSSD61 | 0 | 0 | 2.43E-06 | 1.66E-05 | 0.028 | 0.231 |
| DTU089 | 0.000132769 | 0.0002738 | 8.04E-05 | 0.000198038 | 0.045 | 0.213 |
| Enterobacter | 0.000559614 | 0.00454256 | 0.000799621 | 0.002007442 | 0.000 | 0.001 |
| Enterococcus | 3.31E-05 | 0.000356966 | 2.61E-05 | 8.80E-05 | 0.022 | 0.292 |
| Erysipelatoclostridium | 0.00019156 | 4.18E-05 | 0.000699346 | 0.000391027 | 0.021 | 0.285 |
| Eubacterium | 3.93E-05 | 0.000106675 | 1.43E-05 | 6.94E-05 | 0.020 | 0.275 |
| Faecalibaculum | 0 | 0 | 3.25E-06 | 2.23E-05 | 0.028 | 0.228 |
| Faecalitalea | 0.000143331 | 0.000668535 | 9.80E-05 | 0.000266906 | 0.049 | 0.224 |
| Fenollaria | 4.64E-05 | 1.29E-05 | 0.00015748 | 8.18E-05 | 0.025 | 0.285 |
| Fermentimonas | 0 | 0 | 1.90E-06 | 1.34E-06 | 0.007 | 0.180 |
| Flaviflexus | 0 | 0 | 5.44E-07 | 3.73E-06 | 0.028 | 0.225 |
| Flavobacterium | 0 | 0 | 2.44E-06 | 1.05E-05 | 0.000 | 0.017 |
| Gaiella_sp._EBR4_RS1 | 0 | 0 | 5.54E-07 | 3.80E-06 | 0.028 | 0.222 |
| Gemmatimonas | 0.00000037 | 0.00000037 | 0.00000766 | 0.00000384 | 0.001 | 0.030 |
| Haemophilus | 0.003434445 | 0.009627662 | 0.006607673 | 0.011295466 | 0.017 | 0.241 |
| Holdemania | 0.000298668 | 0.000303603 | 0.000216163 | 0.000249437 | 0.038 | 0.184 |
| Hungatella | 0.003659674 | 0.016400043 | 0.003672584 | 0.011981931 | 0.034 | 0.167 |
| Iamia | 0 | 0 | 8.19E-07 | 5.62E-06 | 0.028 | 0.220 |
| Idiomarina | 2.23E-07 | 2.23E-07 | 6.26E-06 | 3.09E-06 | 0.000 | 0.022 |
| Idiomarina_sp. | 0 | 0 | 8.18E-07 | 5.61E-06 | 0.028 | 0.217 |
| Ignatzschineria | 1.85E-05 | 4.02E-06 | 4.24E-05 | 1.52E-05 | 0.031 | 0.285 |
| Ilumatobacter | 0 | 0 | 2.46E-06 | 9.91E-06 | 0.000 | 0.016 |
| Kaistia | 0 | 0 | 5.41E-07 | 3.71E-06 | 0.028 | 0.215 |
| Klebsiella | 0.000951634 | 0.003918369 | 0.003340321 | 0.007900377 | 0.000 | 0.000 |
| Lachnospiraceae_NK4A136_group | 0.02804618 | 0.034967536 | 0.018785065 | 0.029613015 | 0.031 | 0.157 |
| Lachnospiraceae_UCG_003 | 0.001898727 | 0.008027263 | 0.004242969 | 0.009182856 | 0.005 | 0.085 |
| Lacticaseibacillus | 2.97E-07 | 2.97E-07 | 2.71E-06 | 1.94E-06 | 0.038 | 0.285 |
| Lactiplantibacillus | 6.13E-05 | 1.03E-05 | 0.000200338 | 2.68E-05 | 0.000 | 0.000 |
| Lactococcus | 6.68E-06 | 3.02E-05 | 2.40E-05 | 6.10E-05 | 0.001 | 0.019 |
| Latilactobacillus | 0 | 0 | 3.82E-06 | 2.62E-05 | 0.028 | 0.212 |
| Limosilactobacillus | 5.24E-06 | 2.47E-06 | 3.33E-05 | 1.20E-05 | 0.000 | 0.022 |
| Luteimonas | 6.20E-05 | 7.06E-06 | 0.000145594 | 1.81E-05 | 0.000 | 0.000 |
| Marinobacter | 0 | 0 | 8.12E-07 | 5.57E-06 | 0.028 | 0.210 |
| Micromonospora | 5.17E-07 | 6.80E-06 | 8.15E-06 | 2.50E-05 | 0.000 | 0.005 |
| Microvirga | 0 | 0 | 8.19E-07 | 5.61E-06 | 0.028 | 0.208 |
| Ottowia | 0 | 0 | 2.45E-06 | 1.68E-05 | 0.028 | 0.205 |
| P3OB_42 | 0 | 0 | 1.13E-05 | 5.88E-06 | 0.000 | 0.019 |
| Paeniclostridium | 2.30E-05 | 0.000210061 | 5.97E-05 | 0.000215437 | 0.023 | 0.287 |
| Pajaroellobacter | 0 | 0 | 5.54E-07 | 3.80E-06 | 0.028 | 0.203 |
| Paracoccus | 4.41E-07 | 4.41E-07 | 1.77E-05 | 1.66E-05 | 0.047 | 0.285 |
| Parvibacter | 0 | 0 | 1.91E-06 | 1.31E-05 | 0.028 | 0.201 |
| Paucilactobacillus | 0 | 0 | 1.61E-05 | 6.98E-06 | 0.000 | 0.002 |
| Pediococcus | 1.71E-06 | 8.93E-07 | 1.01E-05 | 7.10E-06 | 0.039 | 0.285 |
| Phocea | 0.00018016 | 0.000345722 | 0.000255056 | 0.000338827 | 0.009 | 0.153 |
| Phormidium_SAG_81.79 | 0 | 0 | 5.46E-07 | 3.74E-06 | 0.028 | 0.199 |
| Phreatobacter | 0 | 0 | 2.45E-06 | 1.68E-05 | 0.028 | 0.197 |
| Planktosalinus | 2.96E-07 | 2.96E-07 | 9.51E-06 | 4.01E-06 | 0.000 | 0.004 |
| Planktothrix_NIVA_CYA_15 | 0 | 0 | 1.62E-06 | 1.11E-05 | 0.028 | 0.195 |
| Polynucleobacter | 0 | 0 | 1.36E-06 | 9.33E-06 | 0.028 | 0.193 |
| Prevotellaceae_UCG_003 | 0 | 0 | 0.00034445 | 0.002042164 | 0.003 | 0.074 |
| Proteiniclasticum | 0 | 0 | 1.09E-06 | 7.45E-06 | 0.028 | 0.191 |
| Pseudofulvimonas | 6.67E-07 | 7.08E-06 | 3.80E-06 | 1.57E-05 | 0.017 | 0.238 |
| Pseudogracilibacillus | 5.69E-06 | 2.24E-05 | 0 | 0 | 0.032 | 0.161 |
| Pseudomonas | 2.44E-05 | 7.44E-05 | 0.000116761 | 0.000690294 | 0.046 | 0.212 |
| Ramlibacter | 0 | 0 | 1.90E-06 | 1.30E-05 | 0.028 | 0.189 |
| Raoultibacter | 7.16E-06 | 3.78E-05 | 2.03E-05 | 7.66E-05 | 0.041 | 0.195 |
| Reyranella | 1.47E-07 | 1.93E-06 | 1.64E-06 | 7.85E-06 | 0.027 | 0.312 |
| Romboutsia | 0.001859699 | 0.000269724 | 0.003411981 | 0.001007198 | 0.036 | 0.285 |
| Roseburia | 0.013899091 | 0.018990862 | 0.023826134 | 0.024965617 | 0.004 | 0.079 |
| Roseiarcus | 0 | 0 | 1.35E-06 | 9.24E-06 | 0.028 | 0.187 |
| Rothia | 0.000240246 | 0.000352783 | 0.0001239 | 0.000200251 | 0.004 | 0.076 |
| S5_A14a | 6.59E-07 | 6.59E-07 | 1.55E-05 | 1.28E-05 | 0.028 | 0.285 |
| Scardovia | 0 | 0 | 3.52E-06 | 1.92E-05 | 0.003 | 0.072 |
| Serratia | 0 | 0 | 4.63E-06 | 3.18E-05 | 0.028 | 0.183 |
| Shinella | 0 | 0 | 9.81E-06 | 6.73E-05 | 0.028 | 0.181 |
| Shuttleworthia | 0.000134893 | 0.000454022 | 0.00012675 | 0.000811725 | 0.045 | 0.212 |
| Sphingobacterium | 0 | 0 | 5.45E-07 | 3.74E-06 | 0.028 | 0.180 |
| Sphingomonas | 7.62E-06 | 2.18E-06 | 0.000120608 | 3.05E-05 | 0.000 | 0.000 |
| Streptococcus | 0.003769619 | 0.00866732 | 0.004684608 | 0.006805765 | 0.024 | 0.295 |
| SWB02 | 0 | 0 | 8.09E-07 | 5.54E-06 | 0.028 | 0.185 |
| Tahibacter | 0 | 0 | 3.27E-06 | 2.24E-05 | 0.028 | 0.178 |
| Terrimonas | 0 | 0 | 5.39E-07 | 3.70E-06 | 0.028 | 0.176 |
| Tissierella | 0 | 0 | 1.09E-06 | 7.47E-06 | 0.028 | 0.175 |
| Turicibacter | 0.000460309 | 0.00274963 | 0.000358628 | 0.000744347 | 0.030 | 0.150 |
| UCG_003 | 0.004717368 | 0.004599469 | 0.003363035 | 0.00341127 | 0.039 | 0.187 |
| UCG_008 | 0 | 0 | 2.66E-05 | 0.000130576 | 0.003 | 0.069 |
| Undibacterium | 0 | 0 | 2.20E-06 | 1.51E-05 | 0.028 | 0.173 |
| Variovorax | 0 | 0 | 2.91E-06 | 2.00E-05 | 0.028 | 0.171 |
| Veillonella | 0.001539016 | 0.005681862 | 0.003535584 | 0.007737063 | 0.035 | 0.172 |
| Weissella | 2.07E-05 | 0.00025084 | 2.35E-05 | 6.46E-05 | 0.000 | 0.013 |

**Supplementary Table S2** The significantly changed metabolites in GDM women vs. Normal pregnant women (n=220).

| MS2 name | RZ | MZ | VIP | P-value | Fold change | Class |
| --- | --- | --- | --- | --- | --- | --- |
| Deoxyribose 5-phosphate | 86.86 | 213.02 | 2.85 | 0.000487341 | 1.26 | Organooxygen compounds |
| Dimethylglycine | 333.11 | 104.07 | 2.03 | 0.000412082 | 1.22 | Carboxylic acids and derivatives |
| Propionic acid | 249.09 | 73.03 | 1.61 | 0.001105946 | 1.17 | Carboxylic acids and derivatives |
| Succinic acid | 403.59 | 117.02 | 1.74 | 0.014901651 | 0.88 | Carboxylic acids and derivatives |
| Malonic acid | 416.97 | 103.00 | 1.67 | 0.016638515 | 1.11 | Carboxylic acids and derivatives |
| Decanoylcarnitine | 207.92 | 316.25 | 1.89 | 0.002549351 | 1.43 | Fatty Acyls |
| Hypoxanthine | 168.92 | 135.03 | 3.50 | 0.000194172 | 0.40 | Imidazopyrimidines |
| L-Glutamic acid | 412.43 | 146.05 | 2.02 | 0.000544918 | 0.71 | Carboxylic acids and derivatives |
| Pyro-L-glutaminyl-L-glutamine | 173.84 | 258.11 | 2.10 | 0.012109647 | 1.12 | Organooxygen compounds |
| Glycerophosphocholine | 393.70 | 258.11 | 3.43 | 0.002728974 | 0.37 | Glycerophospholipids |
| L-Palmitoylcarnitine | 191.77 | 400.34 | 1.96 | 0.027861742 | 0.87 | Fatty Acyls |
| Methylmalonic acid | 196.83 | 117.02 | 2.04 | 0.004069576 | 1.12 | Carboxylic acids and derivatives |
| D-Galactose | 306.19 | 179.06 | 2.57 | 0.016116125 | 1.16 | Organooxygen compounds |
| Isobutyryl-L-carnitine | 270.04 | 232.15 | 1.23 | 0.030262754 | 1.17 | Fatty Acyls |
| D-Ribose | 198.10 | 149.05 | 2.82 | 0.011161879 | 1.20 | Organooxygen compounds |
| D-Malic acid | 424.56 | 133.01 | 1.91 | 0.020892257 | 0.70 | Hydroxy acids and derivatives |
| Fumaric acid | 48.24 | 115.00 | 2.58 | 0.028199303 | 0.12 | Carboxylic acids and derivatives |
| L-Arabinose | 86.25 | 149.05 | 2.87 | 0.004556809 | 1.29 | Organooxygen compounds |
| 4-Acetylbutyrate | 54.15 | 129.06 | 2.22 | 0.000161418 | 1.17 | Fatty Acyls |
| L-Lactic acid | 231.83 | 89.02 | 3.30 | 0.001341584 | 0.74 | Hydroxy acids and derivatives |
| Ethylbenzene | 31.74 | 107.09 | 3.19 | 2.32152E-19 | 2.25 | Benzene and substituted derivatives |
| Norophthalmic acid | 450.66 | 276.12 | 1.45 | 4.50443E-06 | 1.36 | Carboxylic acids and derivatives |
| LysoPE(16:0/0:0) | 217.11 | 454.29 | 2.38 | 0.030392141 | 0.80 | Glycerophospholipids |
| D-Mannose | 283.46 | 179.06 | 2.99 | 6.06527E-07 | 1.34 | Organooxygen compounds |
| L-Malic acid | 469.23 | 133.01 | 2.09 | 0.020491436 | 0.81 | Hydroxy acids and derivatives |
| Niacinamide | 55.62 | 123.05 | 1.02 | 0.002376546 | 0.44 | Pyridines and derivatives |
| Gluconic acid | 331.74 | 195.05 | 2.74 | 0.000223845 | 1.28 | Organooxygen compounds |
| 1,11-Undecanedicarboxylic acid | 272.67 | 243.16 | 1.77 | 0.000323476 | 1.29 | Fatty Acyls |
| LysoPA(16:0/0:0) | 210.77 | 409.24 | 1.75 | 9.37102E-08 | 1.66 | Glycerophospholipids |
| Pyruvic acid | 41.37 | 87.01 | 1.73 | 0.005474559 | 0.70 | Keto acids and derivatives |
| beta-Cryptoxanthin | 29.88 | 552.43 | 1.20 | 0.006201091 | 1.19 | Prenol lipids |
| Gluconolactone | 163.09 | 177.04 | 2.99 | 0.001075155 | 1.25 | Organooxygen compounds |
| D-Glutamic acid | 85.58 | 146.05 | 2.88 | 0.009099556 | 1.23 | Carboxylic acids and derivatives |
| Pyrrolidonecarboxylic acid | 313.28 | 128.04 | 1.84 | 0.042738106 | 0.75 | Carboxylic acids and derivatives |
| Homo-L-arginine | 536.34 | 189.13 | 1.77 | 0.002413791 | 1.52 | Carboxylic acids and derivatives |
| L-Tryptophan | 273.18 | 205.10 | 1.16 | 0.023543815 | 0.77 | Indoles and derivatives |
| LysoPC(20:3(5Z,8Z,11Z)) | 207.19 | 546.35 | 2.12 | 0.004332809 | 0.86 | Glycerophospholipids |
| Citric acid | 90.73 | 191.02 | 2.73 | 0.010540586 | 1.24 | Carboxylic acids and derivatives |
| 4-Hydroxyproline | 353.59 | 130.05 | 1.16 | 0.02054224 | 0.74 | Carboxylic acids and derivatives |
| Sphinganine | 103.78 | 302.31 | 1.78 | 0.008621237 | 0.66 | Organonitrogen compounds |
| Bergapten | 284.15 | 215.03 | 3.80 | 9.12631E-08 | 1.34 | Coumarins and derivatives |
| LysoPC(18:1(9Z)) | 208.50 | 522.36 | 2.13 | 0.01735208 | 0.89 | Glycerophospholipids |
| L-Homoserine | 164.68 | 120.07 | 3.10 | 0.015431544 | 1.21 | Carboxylic acids and derivatives |
| (R)-3-Hydroxybutyric acid | 242.12 | 103.04 | 1.33 | 0.00439615 | 1.45 | Hydroxy acids and derivatives |
| PC(22:6(4Z,7Z,10Z,13Z,16Z,19Z)/P-18:1(11Z)) | 136.54 | 816.59 | 1.94 | 0.040560605 | 1.11 | Glycerophospholipids |
| Isocitric acid | 169.73 | 191.02 | 1.74 | 0.003436385 | 1.46 | Carboxylic acids and derivatives |
| p-Cresol sulfate | 24.50 | 187.01 | 1.32 | 0.021478555 | 1.31 | Organic sulfuric acids and derivatives |
| Ascorbic acid | 85.08 | 175.02 | 3.51 | 0.003218108 | 1.27 | Dihydrofurans |
| 3-Formyl-6-hydroxyindole | 240.86 | 162.06 | 2.67 | 0.00049416 | 0.55 | Indoles and derivatives |
| Glutaric acid | 302.48 | 131.03 | 2.51 | 0.017409362 | 1.19 | Carboxylic acids and derivatives |
| Arbutin | 88.22 | 271.08 | 2.45 | 0.029274707 | 1.40 | Organooxygen compounds |

## Supplementary Figures


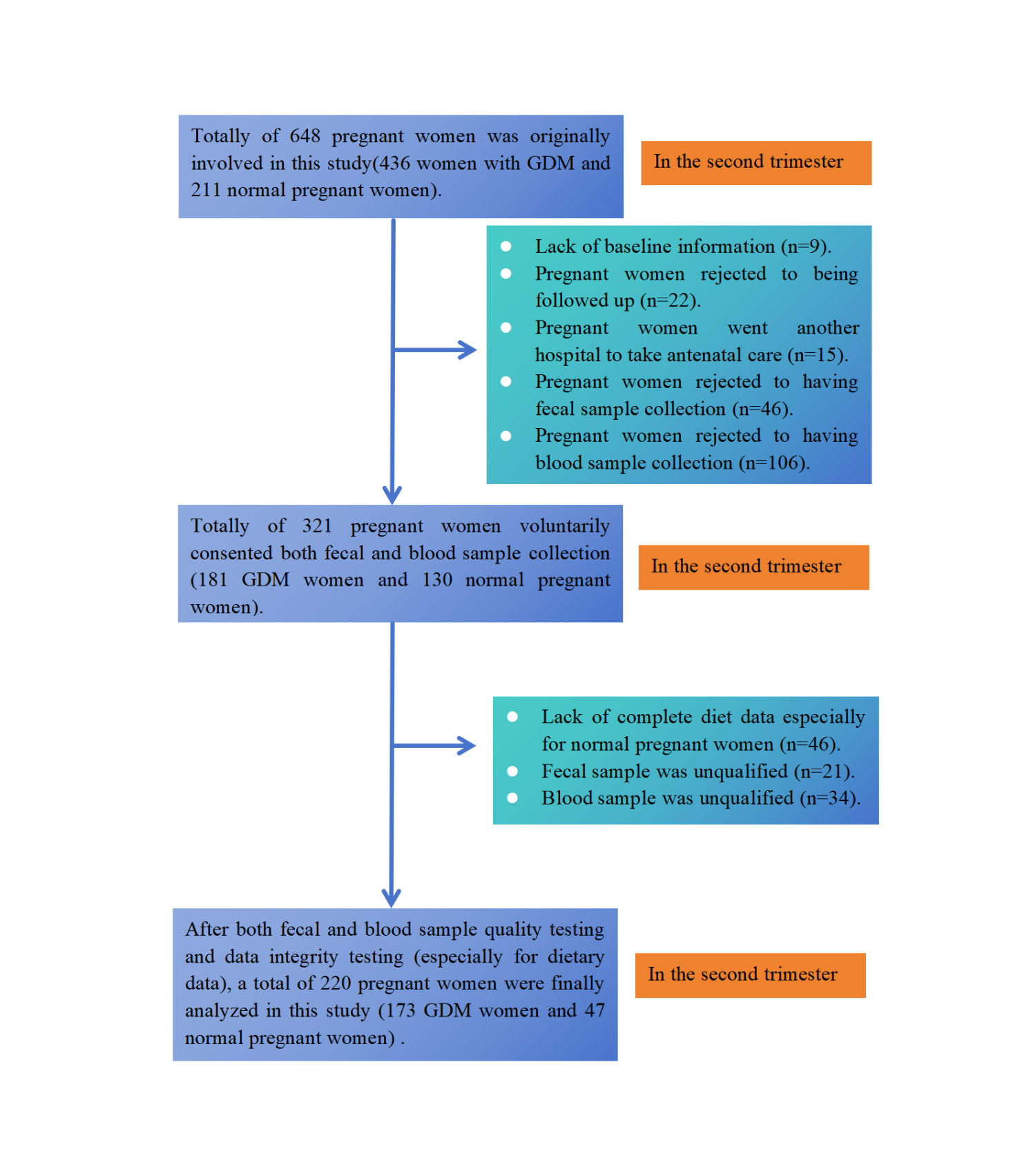


**Supplementary Figure S1.** Inclusion flow of study population.


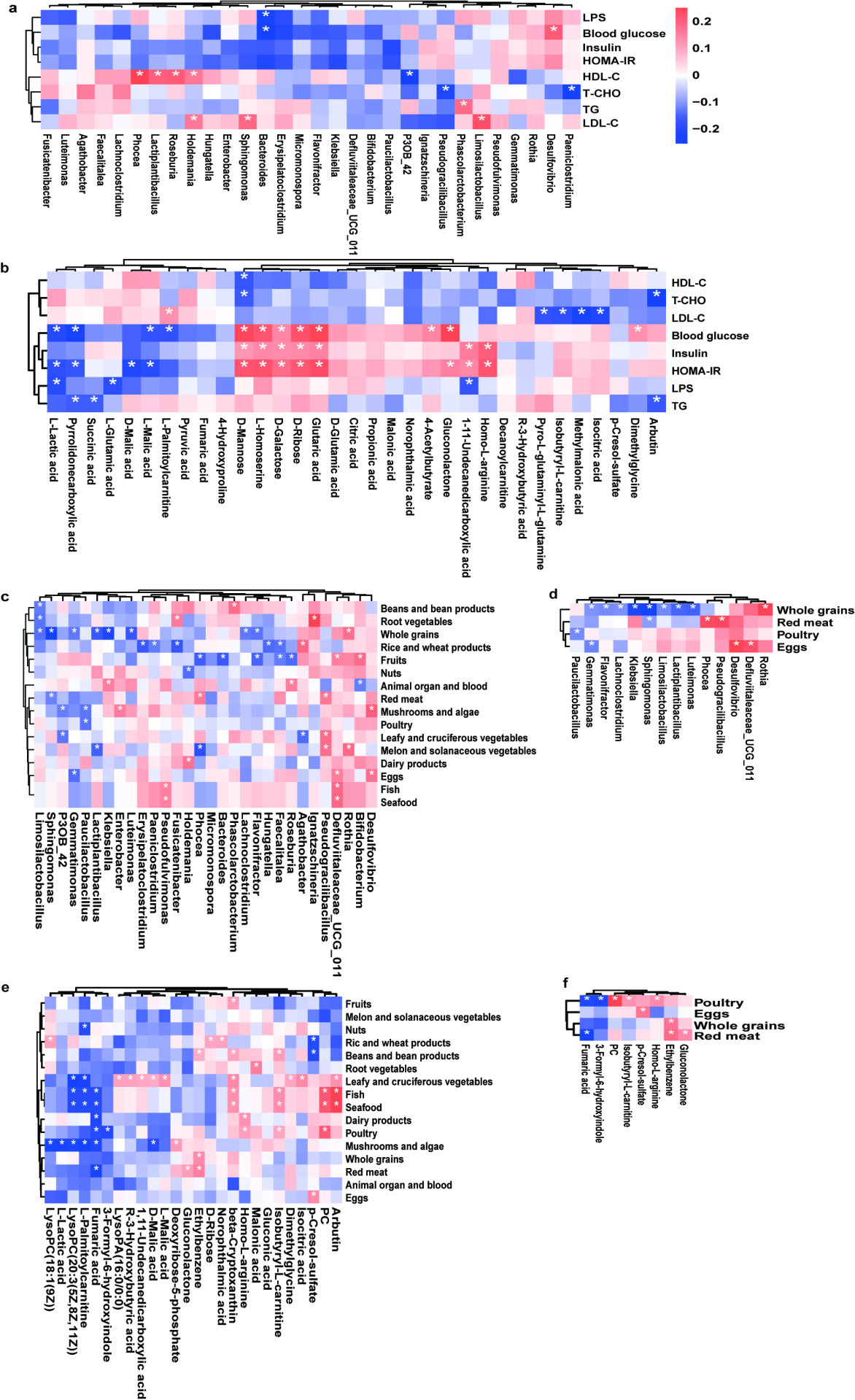


**Supplementary Figure S2.** Spearman’s correlation analysis of plasma parameters, diet, gut microbiota and metabolites in pregnant women with GDM (*P < 0.05).


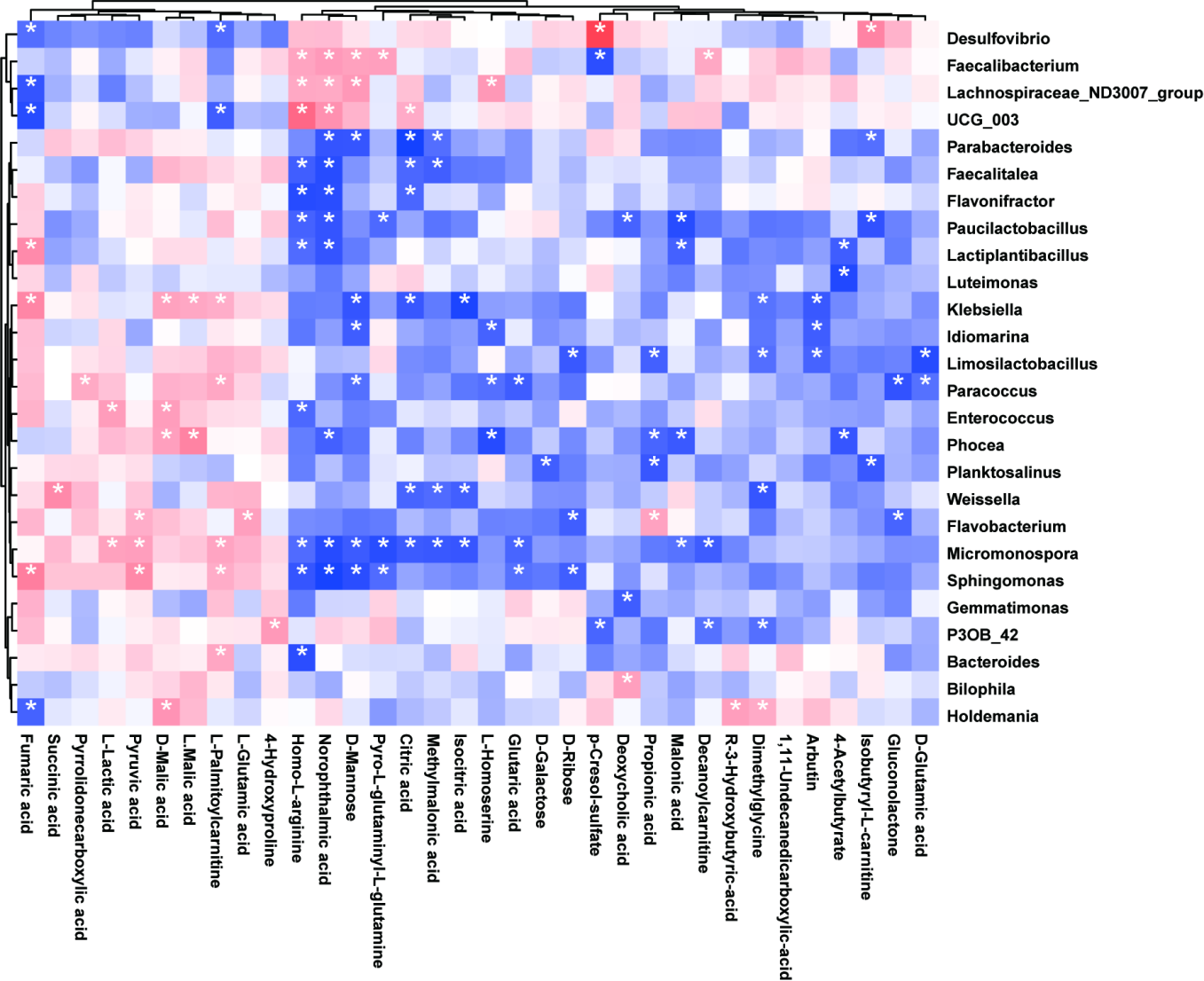


**Supplementary Figure S2.** Spearman’s correlation analysis between gut microbiota and metabolites in pregnant women with GDM (*P < 0.05).
